# Modified score based on revised Tokuhashi score is needed for the determination of surgical intervention in patients with lung cancer metastases to the spine

**DOI:** 10.1186/s12957-019-1738-x

**Published:** 2019-11-18

**Authors:** Zhenyu Cai, Xiaodong Tang, Rongli Yang, Taiqiang Yan, Wei Guo

**Affiliations:** Musculoskeletal Tumor Center, Peking University People’s Hospital, No. 11 Xizhimen South Street, Xicheng District, Beijing, 100044 China

**Keywords:** Modified score, Surgical intervention, Lung cancer metastases to the spine, Predicting survival, Tumor marker, Targeted therapy

## Abstract

**Background:**

Revised Tokuhashi score (RTS) is no longer accurate to predict the survival of patients with lung cancer metastases to the spine. This study is to identify additional prognostic factors in those patients, develop a modified prognostic score based on RTS, and verify the accuracy of the score in prediction.

**Methods:**

Our study included patients with lung cancer metastases to the spine who underwent surgery for spine metastasis. Potential prognostic factors were analyzed. Points were allocated for prognostic factors obtained from survival analyses. A modified score was developed by including prognostic factors and their points to RTS. Accuracy of the modified score was evaluated by comparing the coincidence between predicted and observed survival. Kaplan–Meier analysis and Cox regression models were used. Predictive values of scores for 6-month survival were measured via receiver operating characteristic (ROC) curves.

**Results:**

Targeted therapy and tumor markers were additional independent prognostic factors. In the modified score, 2 and 1 points were allocated to the new evaluation factors. The points for factors based on RTS remained the same, and two prognostic groups were redefined. For group A patients who were predicted to live for less than 6 months, conservative procedures would be recommended. For group B patients who were predicted to live for 6 months or more, palliative surgery would be recommended. When comparing the modified score to RTS, the area under the receiver operating characteristic curve (AUCROC) and accuracy of score were improved.

**Conclusions:**

The modified RTS has improved prognostic accuracy in patients with lung cancer metastases to the spine.

## Introduction

The spine is a common site of bone metastases which easily cause metastatic spinal cord compression (MSCC) [1, 2]. The predicted survival of patients with spinal metastases is a vital factor to determine whether patients should receive spinal surgery. Survival is predicted by several prognostic scoring systems, such as the Revised Tokuhashi score (RTS) [3], Tomita score [4], and Bauer score [5]. RTS is widely used to select patients suitable for surgical decompression and stabilization of the spine. Tokuhashi et al. [3] indicated that RTS is accurate for patients with metastatic spine tumor and observed that the rate of consistency between prognostic and actual survival periods is 82.5%.

However, prognostic accuracy of RTS is controversial when it is utilized to evaluate the prognosis of patients with lung cancer metastases to the spine. Several studies [6–9] on patients with lung cancer have also found that RTS is less reliable in predicting these survivals. Tokuhashi et al. [10, 11] further stated that the usefulness of RTS is insufficient for lung cancer metastases. Its inaccuracy for patients with lung cancer may be attributed to its limitations, that is, studies have included different primary tumors rather than one specific tumor, and the number of each specific primary tumor is few; for example, 48 patients with lung cancer have been included in RTS development [3]. With the introduction of new therapies, such as molecular targeted therapy, these survivals have been improved [12, 13]. As such, RTS may be no longer accurate for predicting the survival of patients with lung cancer metastases to the spine. Hence, a modified system based on RTS is needed.

This study aims to (1) identify additional prognostic factors of patients with lung cancer metastases to the spine, (2) develop a modified prognostic score based on RTS which is specific for determining the surgical intervention of these patients, and (3) verify the accuracy of the modified score.

## Patients and methods

This study was performed after approval was obtained from the institutional review board of our hospital. A total of 140 patients who suffered from lung cancer metastases to the spine and were treated surgically in our institute between March 2010 and March 2017 were retrospectively reviewed. Furthermore, 45 patients were prospectively reviewed between March 2017 and July 2018. Their medical history, image files, and follow-up information were collected and reviewed. The following inclusion criteria were considered: patients who (1) experienced MSCC caused by spine metastasis mass, pathologic fracture, or others; (2) were diagnosed with lung cancer metastasis to the spine as confirmed by pathological examination; (3) received palliative surgery (posterior decompression and internal fixation); (4) died of the disease, or survived and had more than 6 months of follow-up. Patients with the following conditions were excluded: (1) received surgical treatments other than palliative surgery; (2) died by accidents or underlying health problems like coronary heart disease; (3) survived but had less than 6 months of follow-up; and (4) did not have complete follow-up data.

A total of 120 patients were included in the retrospective study. Of these patients, 71 were males and 49 were females, and their mean age was 62 years (range of 27–88 years). After a mean time of 11.8 months (range of 0.3–66 months) of follow-up, 91.7% of the patients (110/120) died of the tumor. The median overall survival (OS) after surgery of the 110 patients who died was 6.8 months (range of 0.3–66 months). In the prospective study, 41 patients, that is, 22 males and 19 females with a mean age of 59 years (range of 28–78 years), were included. After a mean time of 9 months (range of 1–22 months) of follow-up, 70.7% of the patients (29/41) died of the tumor. The median OS after surgery of the 29 patients who died was 9 months (range of 1–22 months).

In addition to known prognostic factors based on RTS, other potential prognostic factors of patients with lung cancer operated for MSCC were derived on the basis of our experience and literature review [3, 5, 14–16]. Characteristic factors were age, gender, history of smoking, and some serological indices, including serum tumor markers (which are related to lung cancer like CEA and AFP), albumin, calcium ion, lactic dehydrogenase, alkaline phosphatase, and erythrocyte sedimentation rate. Disease factors were histologic-type neoplasms (adenocarcinoma versus others) and initial diagnosis of the presence or absence of spinal metastasis. Intervention factors included the adoption of targeted therapy (such as tyrosine kinase inhibitor erlotinib and gefitinib specifically block epidermal growth factor receptor), bisphosphonates, and other adjuvant treatments, including chemotherapy or radiation therapy. Points were allocated for prognostic factors gotten from prognostic factor analyses. A modified score for the prediction of survival was developed on the basis of prognostic factors and their points added to RTS. The accuracy of the modified score was evaluated by comparing the modification of RTS to the original version in the coincidence degree between predicted and observed survival.

All analyses were performed using GraphPad Prism 5.0 (GraphPad Inc., San Diego, CA), SPSS version 18.0 (SPSS, Inc., Chicago, IL) and R-3.3.2 statistical software package. Postoperative survival was estimated through Kaplan–Meier analysis in which death caused by the disease was considered as an event. Survival curves were compared with logrank test results. Cox regression models were used to assess the effects of prognostic variables. Factors with *p* < 0.05 in univariate Cox regression analysis were subjected to the Wald test for multivariate Cox regression analysis. Follow-up time was chosen as the time scale in the Cox regression models. Follow-up time was recorded from the date of the performance of surgery until the date of death or the last follow-up. Differences with *p* < 0.05 were considered significant. Predictive values of the modified score and RTS for 6-month survival were measured via ROC curves.

## Results

**Fig. 1:**
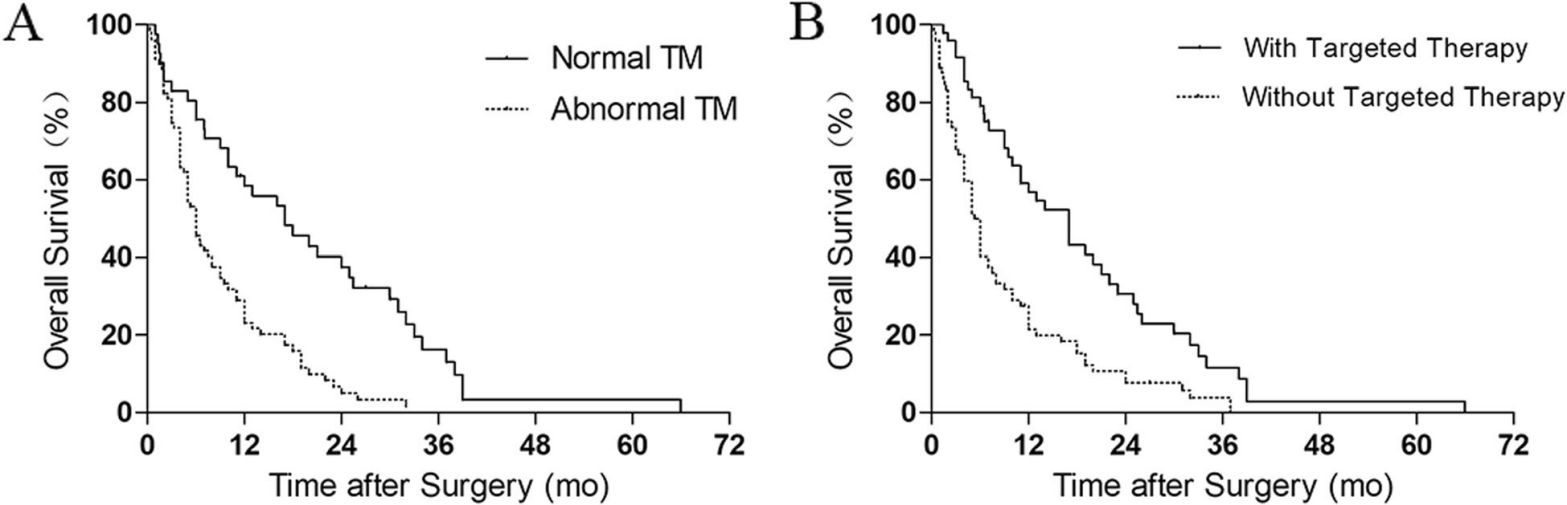
Kaplan–Meier curves of the overall survival of 120 patients with lung cancer metastases to the spine: **a** Patients with normal and abnormal tumor markers (TM; *p* < 0.0001, logrank test). **b** Patients who were and were not subjected to targeted therapy (*p* < 0.0001, logrank test)

The results of the prognostic factor analysis are shown in Table 1. In univariate Cox regression analysis, normal tumor markers of lung cancer, adoption of targeted therapy, and adoption of bisphosphonates increased the probability of good prognosis. In multivariate Cox regression analysis, adoption of targeted therapy and normal tumor markers were found as independent prognostic factors. The prognosis of patients who did not receive targeted therapy was poorer than that of patients who underwent targeted therapy (HR, 2.207; 95% CI, 1.471–3.310). The prognosis of patients with abnormal tumor markers was poorer than that of patients with normal tumor markers (HR, 2.526; 95% CI, 1.620–3.937). Kaplan–Meier analysis revealed the significance of the two factors (Fig. 1).

**Table 1:** Prognostic factor analysis in 120 patients with lung cancer metastases to the spine

Prognostic factor	n*	Kaplan–Meier		Univariate COX regression		Multivariate Cox regression	
		Median OS^Δ^	*p*	HR (95% CI)^◇^	*p*	HR (95% CI) ◇	*p*
Gender							
Female	49	12					
Male	71	6	0.1246	1.346 (0.910–1.991)	0.136		
Age							
≤ 66.5 years	79	10					
> 66.5 years	41	6	0.2953	1.226 (0.828–1.814)	0.309		
History of smoking							
With	90	8					
Without	30	7	0.6074	1.115 (0.727–1.712)	0.618		
Tumor marker							
Normal	41	17					
Abnormal	79	6	< 0.0001	2.557 (1.639–3.992)	0.000	2.526 (1.620–3.937)	0.000
ESR							
Normal	55	11					
Abnormal	65	6	0.7543	1.061 (0.726–1.551)	0.761		
ALP							
Normal	72	8					
Abnormal	48	8	0.4794	1.147 (0.777–1.696)	0.490		
LDH							
Normal	61	12					
Abnormal	59	6	0.0815	1.386 (0.949–2.023)	0.091		
Albumin							
Normal	53	10					
Abnormal	67	6.6	0.6189	1.098 (0.752–1.605)	0.628		
Calcium ion							
Normal	80	7					
Abnormal	40	10	0.3286	0.822 (0.549–1.231)	0.342		
Neoplasms histologic type of lung cancer							
Adenocarcinoma	97	9.5					
Others	23	5	0.5151	1.165 (0.725–1.872)	0.528		
The adoption of targeted therapy							
With	48	17					
Without	72	5.65	< 0.0001	2.231 (1.487–3.347)	0.000	2.207 (1.471–3.310)	0.000
The adoption of bisphosphonates							
With	36	13					
Without	84	6	0.0355	1.533 (1.015–2.315)	0.042	0.904 (0.563–1.451)	0.676
Initial diagnosis because of spinal metastasis or not							
No	39	6					
Yes	81	9	0.9079	0.977 (0.656–1.455)	0.910		
Adoption of adjuvant treatment (including chemotherapy or radiation therapy)							
With	65	10					
Without	55	7	0.2584	1.237 (0.846–1.806)	0.272		

*Number of patients

^Δ^Median overall survival (mo)

^◇^Hazard ratio (95% CI)

*OS*, overall survival; *HR*, hazard ratio; *CI*, confidence interval; *ESR*, erythrocyte sedimentation rate; *ALP*, alkaline phosphatase; *LDH*, lactic dehydrogenase

A scoring system (Table 2) was developed in our study. Adoption of targeted therapy and tumor marker levels were added to RTS. For the adoption and nonadoption of targeted therapy, 2 and 1 points were allocated respectively. For the level of tumor markers, 2 and 1 points were assigned to normal and abnormal results respectively. Points for factors derived from RTS remained the same. The total score ranged from 0 to 14, and two prognostic groups were redefined. Patients in group A (*n* = 58) with scores of 0–8 were predicted to live for less than 6 months (equal to group A in RTS), whereas patients in group B (*n* = 62) with scores of 9–14 were predicted to live for 6 months or more (corresponding to group B and group C in RTS). Conservative procedures and palliative surgery were recommended for patients in groups A and B, respectively.

**Table 2:** Modified surgical treatment score based on the revised Tokuhashi score for patients with lung cancer metastases to the spine

Prognostic factor	Points	No. of patients
General condition (performance status)		
Poor (PS 10–40%)	0	23
Moderate (PS 50–70%)	1	45
Good (PS 80–100%)	2	52
No. of extraspinal bone metastases		
≥ 3	0	34
1–2	1	25
0	2	61
No. of metastases in the vertebral body		
≥ 3	0	63
2	1	20
1	2	37
Metastases to the major internal organs		
Unremovable	0	21
Removable	1	2
No metastases	2	97
Primary site of cancer		
Lung	0	120
Palsy		
Complete (Frankel A B)	0	5
Incomplete (Frankel C D)	1	83
None (Frankel E)	2	32
Tumor markers		
Abnormal	1	79
Normal	2	41
Adoption of targeted therapy		
Without	1	72
With	2	48
Prognostic groups	Total points	
Group A	0–8	58
Group B	9–14	62

**Fig. 2:**
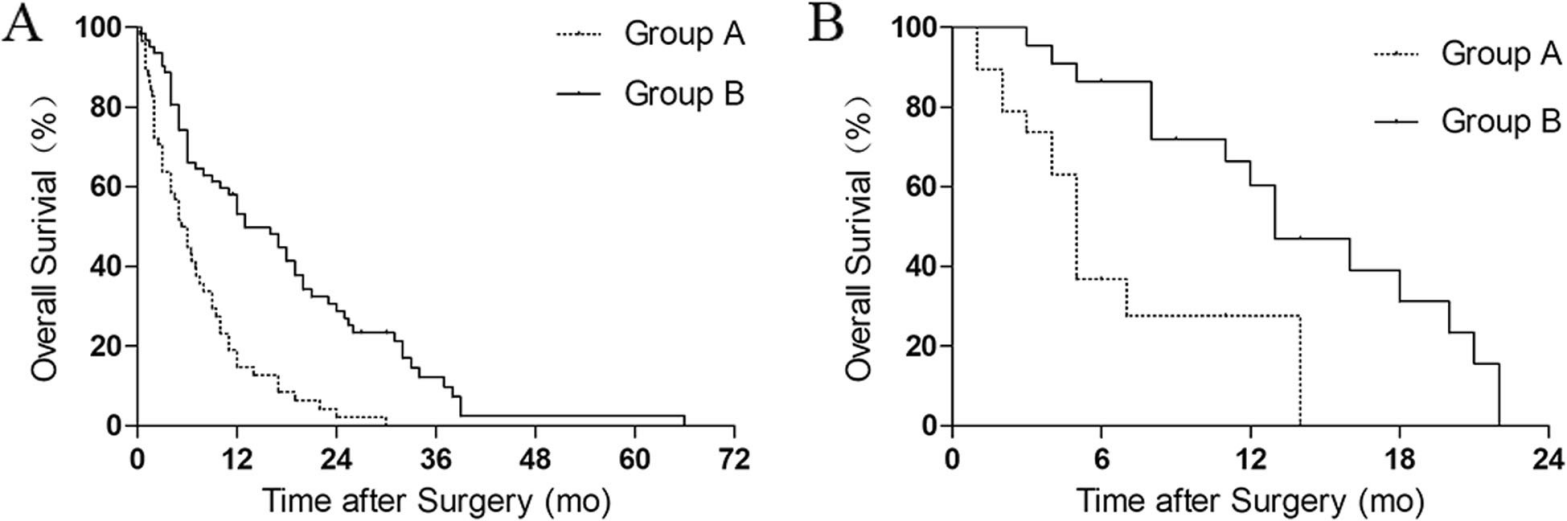
Kaplan–Meier curves of the overall survival of the patients in the two groups based on the modified score of patients with lung cancer metastases to the spine. **a** Retrospective study (*p* < 0.0001, logrank test). Group A: *n* = 58, group B: *n* = 62. **b** Prospective study (*p* = 0.0007, logrank test). Group A: *n* = 19, group B: *n* = 22

For accuracy verification, RTS and the modified score were evaluated in our study. In the retrospective study, no significant difference in survival was found in RTS (compared in Kaplan–Meier analysis, *p* = 0.2155). Predicted and observed survival matched in 36.67% (44 of 120) of patients, including 38.46% (40 of 104) patients in group A (<  6 months) and 25.00% (4 of 16) patients in group B (6–12 months). But the survival of the patients in the two groups of the modified RTS significantly differed when they were compared through Kaplan–Meier analysis (*p* < 0.0001; Fig. 2a). In the modified score, 50.00% (29 of 58) of the patients in group A lived for less than 6 months, and 74.19% (46 of 62) of the patients in group B lived for more than 6 months. For all of the patients in groups A and B, the predicted and observed survival matched in 62.50% (75 of 120). When we referred to 6-month survival, the modified score was more accurate with an AUCROC of 0.629 than RTS with an AUCROC of 0.511.

In the prospective study, the modified RTS indicated that 19 and 22 patients could be classified in groups A and B. Kaplan–Meier analysis demonstrated that the two groups significantly varied (*p* = 0.0007; Fig. 2b). 63.16% (12 of 19) of the patients in group A lived for less than 6 months, and 86.36% (19 of 22) of the patients in group B lived for more than 6 months. For all of the patients, the predicted and observed survival matched in 75.61% (31 of 41). When we referred to 6-month survival, the modified score was accurate with an AUCROC of 0.765 to RTS with an AUCROC of 0.523.

## Discussion

RTS is widely used to predict the survival of patients with spinal metastases and to recommend whether or not these patients should receive spinal surgery [3, 11, 17]. However, RTS is not specific for patients with lung cancer metastases to the spine. With the introduction of new therapeutic methods, such as molecular targeted therapy, the survival of patients with lung cancer has been enhanced [13, 18]. Consequently, it is unreasonable to predict these survivals with RTS. Hence, we performed this study to determine the independent prognostic factors in addition to general factors in RTS, to develop a prognostic score based on RTS and suitable for patients with lung cancer metastases to the spine, and to check the validity of the modified version.

In our study, adoption of targeted therapy and normal tumor markers were identified as additional specific prognostic factors. Several studies [10, 19, 20] have reported that molecularly targeted drug is effective against nonsmall cell lung cancer (NSCLC) and prolongs the survival of patients with NSCLC, suggesting that prognostic scoring systems should be changed. Uei et al. [10] found that the mean post-treatment survival periods are 5.1 and 9.3 months before and after molecularly targeted drugs are included in treatment strategies for lung cancer, respectively. In our series, 48 of 120 patients adopting targeted therapy had a median OS of 17 months, which was longer than the median OS of 5.65 months of 72 patients who did not undergo targeted therapy. The result of this clinical study confirmed the status of targeted therapy as a specific prognostic factor. Several studies [21, 22] have also reported that high pretreatment serum levels of lung cancer biomarkers are associated with the poor outcome of patients with NSCLC. Fiala et al. [22] observed that the median OS of patients with high CEA is 8.6 months compared with that of patients with low CEA (16.1 months). In our series, the median OS of the patients with abnormal tumor markers was 6 months, while the median OS of the patients with normal tumor markers was 17 months. Our result confirmed that the serum tumor markers of lung cancer were the specific prognostic factor of these patients. Hence, the two prognostic factors were added to RTS in our study.

When Crnalic et al. [23] allocated points for prognostic factors after COX analysis, the proportion of the score for prognostic parameters was based on the clinical influencing value of factor for patients’ survival rather than statistical analysis results only. As a result, by adding targeted therapy adoption to the score, we wanted to increase awareness that the eligibility to targeted therapy represents the most important factor determining survival in this patients’ population. Therefore, we gave targeted therapy adoption the same weight in the modified score to other factors like performance status and visceral metastasis. Besides, tumor marker is the strong predictor of survival in lung cancer patients with spinal metastases. Hence, we gave tumor markers the same weight in the score like other parameters. When it comes to how to score points for prognostic factors, there were two aspects to clarify. First, we considered that RTS underestimated the survival of those patients, which might be caused by the low basic score (0 point) of the lung cancer in RTS. And the low basic score trend resulted in the low total score trend. In order to correct the trend, we increased the basic score of lung cancer patients to 2 points while adding two prognostic factors into the score. And we average 1 point to cases with abnormal tumor marker or no adaption of the targeted therapy. In addition, the weight of tumor markers and adoption of targeted therapy were related and consistent with the original prognosis factors in the RTS. So we assigned 2 points to normal tumor markers and adoption of targeted therapy.

The groups were redefined to predict the survival, that is, three groups for treatment selection were modified into two groups. Three treatment selections based on RTS [3] are recommended for patients with spinal metastases: conservative treatment, palliative surgery, and excisional surgery. The prognosis of patients with lung cancer is poor, so conservative treatment or palliative surgery is usually recommended for patients who have lung cancer metastases to the spine but are not candidates for aggressive excisional surgery [24, 25]. Although the survival of patients with lung cancer has increased, patients in advanced stages do not belong to the group with long-term survival. In our study, the median OS was 6.8 months, indicating that excisional surgery was unsuitable for these patients. What’s more, the primary purpose of modified RTS is to identify patients who are suitable to receive spinal surgery. Then, as for patients who need surgical treatment, which type of surgery is optimal depends on the experience of surgeon and the result of MDT rather than just score system. As such, two groups were set at a cutoff point of 6 months to provide two treatment options, namely, conservative treatment and palliative surgery, which were sufficient to meet the requirements for clinical practice.

The accuracy of our modified score was enhanced to predict the survival of patients with lung cancer metastases to the spine. Tokuhashi et al. [10, 11] stated that the usefulness of RTS is insufficient for lung cancer metastases. Gakhar et al. [6] reported an RTS accuracy rate of 44% for their lung cancer subgroup. Likewise, Tan et al. [7] observed that the predicted survival of 75 of 180 patients (41.7%) is correlated with their actual survival via RTS and indicated that Tomita, modified Bauer, and Oswestry scores are inaccurate for patients with lung cancer. In our study, RTS underestimated patients’ survival, suggesting that RTS is not appropriate for guiding clinical decisions in these patients. However, the AUCROC was improved when we applied the modified score based on a large sample size, specific for patients with lung cancer spinal metastases. At the same time, the accuracy in retrospective and prospective validation reached 62.5% and 75.61%. The accuracy of the modified version for prediction was improved when compared with 38.46% of RTS.

This study has some limitations. First, the modified score was developed on the basis of retrospective data. Second, this study included patients who underwent surgery, and whether patients could receive spinal surgery was based on the decision of a surgeon; that is, patients undergoing spinal surgery usually had a good performance status. Third, this study was a regional research in the Chinese people. Besides, further prospective cases should be considered to verify the accuracy of this modified score. Despite these limitations, we conducted a relatively large-scale study on patients with lung cancer metastases to the spine. We could obtain important information as a basis for determining patients who should receive spinal surgery.

## Conclusions

In conclusion, adoption of targeted therapy and normal tumor markers are prognostic factors for patients with lung cancer metastases to the spine. These two prognostic factors are added to RTS, and a score specific for these patients is developed. The modified RTS has improved prognostic accuracy, thereby helping clinicians select appropriate treatments for these patients.

## Data Availability

Please contact the author for data requests.

## Abbreviations

MSC: Metastatic spinal cord compression
OS: Overall survival
ROC: Receiver operating characteristic
AUCROC: Area under the receiver operating characteristic curve
RTS: Revised Tokuhashi score
